# Anesthetic Management of One-Lung Ventilation in Thoracic Trauma: A Retrospective Observational Cohort Study

**DOI:** 10.7759/cureus.111563

**Published:** 2026-06-26

**Authors:** Binayak Deb, Tridha Majumdar, Shreya Neogy, Abhishek Singh, Yudhyavir Singh, Riniki Sarma, Sharmishtha Pathak, Kapil Dev Soni, Richa Aggarwal, Chhavi Sawhney, Babita Gupta

**Affiliations:** 1 Division of Trauma Anesthesia and Critical Care, All India Institute of Medical Sciences, New Delhi, IND

**Keywords:** acute respiratory distress syndrome, anesthesia management, bronchial blocker, double-lumen tube, one-lung ventilation, thoracic trauma

## Abstract

Background

One-lung ventilation (OLV) in acute thoracic trauma presents physiological and technical challenges distinct from elective thoracic anesthesia. Preexisting hypoxemia, hemodynamic instability, and the inflammatory milieu of polytrauma increase susceptibility to ventilator-induced lung injury and postoperative pulmonary complications (PPCs). Data on anesthetic management, technique selection, and outcomes in this population remain limited. We report our experience with OLV in 117 consecutive trauma patients, characterizing clinical practice patterns and identifying predictors of acute respiratory distress syndrome (ARDS).

Methods

We conducted a retrospective observational cohort study of all trauma patients requiring OLV at the Jai Prakash Narayan Apex Trauma Centre, All India Institute of Medical Sciences, New Delhi, over six years (January 2020 to December 2025). Patient demographics, mechanism of injury, injury patterns, preoperative physiology, OLV technique, intraoperative ventilation parameters, surgical approach, and postoperative outcomes were systematically recorded. Subgroup analyses compared blunt vs penetrating injury, OLV techniques, and video-assisted thoracoscopic surgery (VATS) vs open thoracotomy. Predictors of ARDS were identified through univariate and multivariate binary logistic regression.

Results

The mean patient age was 35.8 ± 13.1 years; 93.2% were male. Road traffic accidents (52.1%) and blunt chest trauma (70.1%) predominated. Double-lumen tubes were used in 50.4% of cases. Intraoperative hypoxia occurred in 48.3% of patients, and ARDS developed in 23.9%. VATS was associated with a significantly lower ARDS rate (10.3% vs 36.2%; P = 0.001) and reduced intraoperative blood transfusion (13.8% vs 31.0%; P = 0.026) compared with open thoracotomy. On multivariate analysis, the principal independent predictors of ARDS were preoperative mechanical ventilation (adjusted OR (aOR) 5.26; 95% CI 1.21-22.75; P = 0.026), maximum intraoperative fraction of inspired oxygen (FiO₂) (aOR 1.05 per percentage point; 95% CI 1.01-1.09; P = 0.021), and surgical approach, with VATS strongly protective relative to open thoracotomy (aOR 0.13; 95% CI 0.04-0.48; P = 0.002). The model demonstrated good discrimination (area under the receiver operating characteristic curve = 0.850) and calibration (Hosmer-Lemeshow P = 0.905).

Conclusions

OLV in thoracic trauma is associated with frequent perioperative hypoxia and PPCs, with preoperative mechanical ventilation, higher intraoperative FiO₂ requirements, and open thoracotomy independently associated with increased ARDS risk, whereas VATS was associated with a lower incidence of ARDS.

## Introduction

One-lung ventilation (OLV) is an essential component of thoracic anesthesia, facilitating surgical exposure and lung isolation during a variety of thoracic procedures [1]. In acute thoracic trauma, however, OLV is particularly challenging because patients frequently present with pulmonary contusion, hemorrhage, aspiration, hypoxemia, hemodynamic instability, and associated injuries that compromise respiratory reserve [2,3]. These factors increase susceptibility to intraoperative hypoxia, ventilator-induced lung injury (VILI), and postoperative pulmonary complications (PPCs), including acute respiratory distress syndrome (ARDS).

The spectrum of thoracic trauma procedures requiring OLV ranges from emergency interventions for pulmonary hemorrhage and airway injury to semi-elective procedures such as retained hemothorax evacuation, empyema surgery, diaphragmatic repair, and retrieval of retained intrathoracic foreign bodies [4,5]. Lung isolation may be achieved using double-lumen tubes (DLTs), bronchial blockers (BBs), or endobronchial intubation (EI), with device selection influenced by airway anatomy, urgency, cervical spine considerations, and anticipated postoperative ventilatory requirements [6-9].

Although lung-protective ventilation strategies during OLV have been associated with improved outcomes in elective thoracic surgery [10-12], their effectiveness in trauma patients remains uncertain because injured lungs are already primed for inflammatory injury. Moreover, the need for higher inspired oxygen concentrations to counter shunt physiology and pulmonary dysfunction may further contribute to oxidative injury and subsequent ARDS [13,14]. Consequently, perioperative hypoxia and PPCs remain major determinants of morbidity in thoracic trauma patients requiring OLV.

Despite the clinical importance of this population, data characterizing OLV practices and outcomes in thoracic trauma remain limited. Most available evidence originates from elective thoracic surgical cohorts, and comparative data regarding lung isolation techniques in trauma-specific settings are scarce [7,10]. Consequently, the optimal anesthetic approach and the perioperative factors influencing pulmonary outcomes in thoracic trauma remain incompletely defined.

This study aimed to evaluate anesthetic management practices and perioperative outcomes among thoracic trauma patients undergoing OLV at a Level I trauma center. The primary objective was to identify independent predictors of postoperative ARDS. Secondary objectives were to compare the performance and clinical outcomes of different lung isolation techniques, assess outcomes according to mechanism of injury and surgical approach, and characterize perioperative pulmonary complications, including intraoperative hypoxia. We hypothesized that patient physiological status and modifiable perioperative factors would have a greater influence on postoperative pulmonary outcomes than the choice of lung isolation device itself.

## Materials and methods

Ethical approval

This study was approved by the Institute Ethics Committee of All India Institute of Medical Sciences (AIIMS), New Delhi (AIIMSA6166/06.03.2026, RP-34/2026). Informed consent was waived as per committee guidance given the retrospective nature of the study. The manuscript was prepared in accordance with the Strengthening the Reporting of Observational Studies in Epidemiology (STROBE) guidelines [15].

Study design and setting

We conducted a single-center, retrospective observational cohort study at the Jai Prakash Narayan Apex Trauma Centre, AIIMS New Delhi, which serves as a Level I trauma center with an annual footfall exceeding 10,000 trauma patients. All patients requiring OLV for thoracic trauma surgery between January 1, 2020 and December 31, 2025 were identified through institutional registry searches, operating theater records, and electronic medical records (EMRs) and were included consecutively. A structured, prespecified pro forma was used by two independent investigators to abstract data systematically from the perioperative and ICU records.

Study population

**Figure 1:**
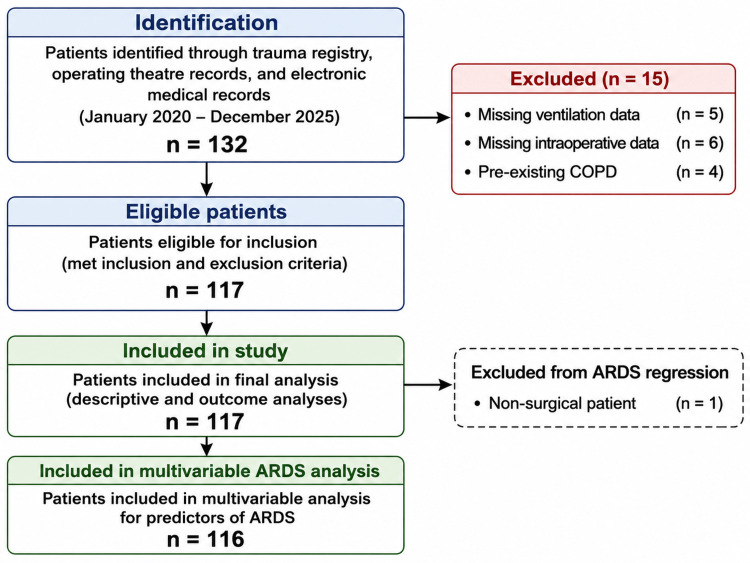
Cohort flow diagram ARDS, acute respiratory distress syndrome; COPD, chronic obstructive pulmonary disease

Patients aged ≥16 years with blunt or penetrating chest trauma as the primary indication for surgery who underwent thoracic surgery requiring pleural cavity access and OLV were eligible for inclusion. A lower age threshold of 16 years was applied because adolescents in this age range are managed under adult trauma and anesthesia protocols at our apex trauma center; two patients aged 16 and 17 years were included on this basis. Patients with preexisting chronic lung disease requiring long-term home oxygen supplementation or chronic ventilatory support, and those for whom key intraoperative or ICU ventilation data were unavailable, were excluded. To minimize selection bias, all consecutive patients undergoing OLV for thoracic trauma between January 2020 and December 2025 were identified through institutional trauma registries, operating theater records, and EMRs. A total of 132 patients were screened for eligibility. Fifteen patients were excluded because of missing key ventilation data (n = 5), incomplete intraoperative records (n = 6), or preexisting chronic obstructive pulmonary disease requiring exclusion according to the predefined study criteria (n = 4). The final study cohort therefore comprised 117 consecutive patients. No patients were excluded based on outcome status, and no sampling strategy was employed (Figure 1).

Data collection

Data were extracted retrospectively from existing medical records covering four perioperative time points: (1) preoperative, from anesthesia pre-assessment notes and ICU charts; (2) intraoperative, from anesthesia records and nursing documentation; (3) immediate postoperative, from recovery and ICU admission notes; and (4) ICU course, from daily progress documentation, respiratory therapy records, and radiological reports. All data abstraction was performed independently by two investigators using a structured pro forma, with discrepancies resolved by consensus and cross-verification against the source EMR entries.

Baseline characteristics recorded included age, weight, sex, mechanism of injury, and injury patterns. Injury severity was quantified using the Injury Severity Score (ISS) and the Thoracic Trauma Severity Score (TTSS). Preoperative physiological status variables included mechanical ventilation status and duration, hemodynamic parameters, oxygenation indices (peripheral oxygen saturation, SpO₂; FiO₂ requirement), arterial blood gas parameters, lactate levels, and vasopressor use. Airway assessment encompassed both anatomical difficulty (Mallampati class, mouth opening, cervical mobility, and airway anatomy) and physiological difficulty (hemodynamic instability, hypoxia, and respiratory distress).

Anesthetic management and OLV technique

Because of the heterogeneous nature of thoracic trauma, decisions regarding lung isolation devices, ventilatory adjustments, rescue maneuvers, and surgical approaches were individualized by the attending anesthesiology and surgical teams rather than governed by a standardized institutional protocol. The indication for OLV, the location of OLV initiation (emergency department, ICU, or operating room), and the device used (DLT, BB, or deliberate EI) were recorded. OLV confirmation time (time from device placement to fiberoptic verification of correct position) and OLV duration were recorded from intraoperative timesheets. First-technique failure was defined as the inability to achieve adequate lung isolation, necessitating a change in device or technique.

Ventilation parameters documented throughout OLV included tidal volume (mL/kg predicted body weight), PEEP (cmH₂O), peak airway pressure, driving pressure, and FiO₂. Application of continuous positive airway pressure (CPAP) to the nondependent lung was recorded. Hemodynamic data captured included heart rate, blood pressure, vasopressor requirement and dose, blood transfusion, and peak intraoperative lactate. Surgical approach (VATS vs open thoracotomy) and VATS-to-open conversion were documented. Regional analgesia modalities, such as thoracic epidural, erector spinae plane block (ESPB), and serratus anterior plane block (SAPB), were recorded.

Aims, hypothesis, and outcomes

The primary aim of this study was to identify independent perioperative predictors of postoperative ARDS in adult thoracic trauma patients managed with OLV, with the working hypothesis that surgical approach and modifiable intraoperative factors, rather than the choice of lung isolation device per se, would be the dominant determinants after adjustment for injury severity.

Primary Outcome

The primary outcome was the development of postoperative ARDS during the index hospital admission, defined according to the Berlin criteria (bilateral infiltrates on chest imaging, PaO₂/FiO₂ ratio <300 mmHg with PEEP ≥5 cmH₂O, onset within one week of a clinical insult, and not fully explained by cardiac failure or fluid overload) [16].

Secondary Outcomes

These were organized into four domains: (i) performance of lung isolation techniques: first-attempt success of the chosen device, time to confirm OLV by fiberoptic bronchoscopy, OLV duration, achievable ventilation parameters (tidal volume, PEEP, peak airway pressure, driving pressure, FiO₂), and intraoperative hypoxia (SpO₂ <90% at any point during OLV); (ii) PPCs: hospital-acquired or ventilator-associated pneumonia (HAP/VAP), ICU hypoxia, intraoperative bronchospasm, and requirement for therapeutic bronchoscopy; (iii) healthcare resource utilization: postoperative mechanical ventilation days, ICU length of stay (LOS), and hospital LOS; and (iv) effect modification by subgroup: comparisons stratified by mechanism of injury (blunt vs penetrating), surgical approach (VATS vs open thoracotomy), and lung isolation technique (DLT vs BB vs EI).

Statistical analysis

Normality was assessed using the Shapiro-Wilk test together with inspection of distribution plots and descriptive statistics. Variables demonstrating non-normal distributions were summarized using median and IQR and analyzed using nonparametric statistical tests. Continuous normally distributed variables are reported as mean ± SD. Categorical variables are expressed as counts and percentages. Between-group comparisons used the Mann-Whitney U test (two groups, continuous) or Kruskal-Wallis test (three or more groups), and the chi-square test or Fisher’s exact test (categorical), as appropriate.

Predictors of ARDS were assessed using univariate binary logistic regression, with associations expressed as ORs and 95% CIs. Variables considered for multivariable logistic regression were selected on the basis of clinical relevance and/or a univariate association with ARDS at P < 0.20. Patients with missing key ventilation or intraoperative data were excluded prior to analysis because these variables were essential to the primary study objectives. No imputation procedures were performed. Covariates entered into the final multivariable model were assessed for clinical plausibility and collinearity before model construction. Model discrimination was assessed using the area under the receiver operating characteristic curve (AUROC), and calibration was evaluated using the Hosmer-Lemeshow goodness-of-fit test. Explained variation was additionally quantified using the Nagelkerke R². Spearman rank-order correlation was used to assess relationships between ISS, peak lactate, OLV duration, and continuous outcome measures.

A two-tailed P value of <0.05 was considered statistically significant. All analyses were performed using IBM SPSS Statistics for Windows, version 29.0 (released 2022; IBM Corp., Armonk, NY, USA) and R version 4.3.2 (R Foundation for Statistical Computing, Vienna, Austria). As this was a complete enumeration of all eligible cases during the study period, no a priori sample size calculation was performed.

## Results

Patient characteristics

During the study period (2020-2025), 117 consecutive trauma patients who underwent OLV for thoracic trauma at our institution met the inclusion criteria (Figure 1). The cohort was predominantly male (n = 109, 93.2%), with a mean age of 35.8 ± 13.1 years (range 16-77). Road traffic accidents accounted for 52.1% of cases, followed by assault (25.6%) and falls from height (16.2%). Blunt chest trauma was the dominant mechanism (70.1%); penetrating injury accounted for 29.9%. Injury severity was moderate to severe overall, with a median ISS of 21 (IQR 16-27) and a median TTSS of 12 (IQR 10-15). Forty-three patients (36.8%) were already mechanically ventilated at the time of assessment. Physiological difficulty in airway management was present in 53.8% of patients; anatomical difficulty was noted in 26.5%. The median time from injury to surgery was seven days (IQR 3-12); 16 patients (13.7%) underwent surgery on the day of injury (Table 1).

**Table 1:** Baseline demographic, injury, and preoperative characteristics of the study participants (N = 117) Data are presented as mean ± SD, median (IQR), or frequency, n (%). ISS: Injury Severity Score; TTSS: Thoracic Trauma Severity Score

Parameter	Variable	Value
Demographics	Age (years), mean ± SD (range)	35.8 ± 13.1 (16-77)
	Weight (kg), mean ± SD (range)	71.8 ± 12.8 (45-110)
	Male sex, n (%)	109 (93.2%)
Mechanism of injury	Road traffic accident, n (%)	61 (52.1%)
	Assault, n (%)	30 (25.6%)
	Fall from height, n (%)	19 (16.2%)
	Other, n (%)	7 (6.0%)
Injury pattern	Blunt chest trauma, n (%)	82 (70.1%)
	Penetrating chest trauma, n (%)	35 (29.9%)
Injuries sustained	Isolated chest injury, n (%)	68 (58.1%)
	Polytrauma, n (%)	49 (41.9%)
Injury severity	ISS, median (IQR)	21 (16-27)
	ISS, mean ± SD (range)	22.7 ± 11.4 (9-75)
	TTSS, median (IQR; range)	12 (10-15; 4-21)
Preoperative status	On mechanical ventilation, n (%)	43 (36.8%)
	Ventilator days if ventilated, median (IQR)	5 (0-10)
	Best SpO₂ (%), median (IQR)	98 (97-100)
	Maximum FiO₂ requirement (%), median (IQR)	40 (30-50)
	Physiologically difficult airway, n (%)	63 (53.8%)
	Anatomically difficult airway, n (%)	31 (26.5%)
Surgical timing	Time from injury to surgery (days), median (IQR)	7 (3-12)
	Immediate surgery (day 0), n (%)	16 (13.7%)

Anesthetic and operative management

The most common indication for OLV was evacuation of retained hemothorax (n = 72, 61.5%), followed by damage control surgery (n = 15, 12.8%), surgical rib fixation (n = 13, 11.1%), and retained foreign body removal (n = 9, 7.7%). DLTs were employed in 59 patients (50.4%), BB in 46 (39.3%), and EI in 12 (10.3%). The first OLV technique failed in eight patients (6.8%), all initially attempted with a DLT; rescue strategies were a BB in seven patients and EI in one patient.

Median OLV confirmation time was 12 minutes (IQR 9-15). EI was confirmed most rapidly (median four minutes, IQR 3-5) compared with BB (10 minutes, IQR 7-14) and DLT (15 minutes, IQR 12-15; Kruskal-Wallis P < 0.001). OLV duration differed significantly by technique (P < 0.001), with EI used predominantly for damage control procedures (median 13 minutes, IQR 12-18) and DLT and BB supporting more prolonged reconstructive operations (medians 50 and 48 minutes, respectively). Protective ventilation was applied throughout: median tidal volume 5 mL/kg predicted body weight (IQR 5-5), PEEP 5 cmH₂O (IQR 5-7), peak airway pressure 30 cmH₂O (IQR 26-36), and driving pressure 14 cmH₂O (IQR 12-16). CPAP was applied to the nondependent lung in 14 patients (12.0%). VATS and open thoracotomy were performed in equal proportions (n = 58 each), with nine of the VATS cases (15.5%) requiring intraoperative conversion to open thoracotomy. Epidural analgesia was employed in 39.3% of patients and regional fascial plane techniques (ESPB or SAPB) in 15.4% (Table 2).

**Table 2:** Anesthetic and operative management details (N = 117) Data are presented as median (IQR) or frequency, n (%). BB: bronchial blocker; CPAP: continuous positive airway pressure; DLT: double-lumen tube; EI: endobronchial intubation; ESPB: erector spinae plane block; OLV: one-lung ventilation; PBW: predicted body weight; PEEP: positive end-expiratory pressure; SAPB: serratus anterior plane block; SSRF: surgical stabilization of rib fractures; VATS: video-assisted thoracoscopic surgery

Parameter	Variable	Value
Indication for OLV	Retained hemothorax, n (%)	72 (61.5%)
	Damage control surgery, n (%)	15 (12.8%)
	Rib fixation (SSRF), n (%)	13 (11.1%)
	Retained foreign body, n (%)	9 (7.7%)
	Decortication/empyema, n (%)	4 (3.4%)
	Other, n (%)	4 (3.4%)
Lung isolation	DLT, n (%)	59 (50.4%)
	BB, n (%)	46 (39.3%)
	EI, n (%)	12 (10.3%)
	Failed first technique, n (%)	8 (6.8%)
	OLV confirmation time (min), median (IQR)	12 (9-15)
	OLV duration (min), median (IQR)	44 (30-60)
Ventilator settings during OLV	Tidal volume (mL/kg PBW), median (IQR)	5 (5-5)
	PEEP (cmH₂O), median (IQR)	5 (5-7)
	Peak airway pressure (cmH₂O), median (IQR)	30 (26-36)
	Driving pressure (cmH₂O), median (IQR)	14 (12-16)
	Maximum intraoperative FiO₂ (%), median (IQR)	50 (50-70)
	CPAP to nondependent lung, n (%)	14 (12.0%)
Intraoperative hemodynamics	Vasopressor requirement, n (%)	34 (29.1%)
	Blood transfusion, n (%)	26 (22.2%)
	Peak lactate (mmol/L), median (IQR)	1.3 (1.0-2.0)
Surgical approach	VATS, n (%)	58 (49.6%)
	Open thoracotomy, n (%)	58 (49.6%)
	Nonsurgical (excluded from regression), n (%)	1 (0.9%)
	Conversion VATS→ open, n (% of VATS)	9 (15.5%)
Regional analgesia	Epidural, n (%)	46 (39.3%)
	ESPB/SAPB, n (%)	18 (15.4%)
	None, n (%)	53 (45.3%)

Intraoperative and postoperative outcomes

Intraoperative hypoxia (SpO₂ <90%) occurred in 56 patients (48.3%) and bronchospasm in 22 (19.0%). The rate of intraoperative hypoxia differed across OLV techniques (DLT 22/59, BB 26/45, EI 8/12; χ²(2) = 6.11, P = 0.047). Forty-three patients (36.8%) were extubated on the operating table; 76 (65.0%) required postoperative ICU admission.

ARDS developed in 28 patients (23.9%), and HAP/VAP in 32 (27.4%). Among the 76 patients admitted to the ICU, hypoxia was observed in 43 (56.6%) and therapeutic bronchoscopy was required in 29 (38.2%). Median ICU LOS was five days (IQR 2-15; mean 10.8 ± 13.4 days), and median hospital LOS was 13 days (IQR 9-26; mean 20.0 ± 16.7 days) (Table 3).

**Table 3:** Intraoperative and postoperative outcomes (N = 117) Data are presented as mean ± SD, median (IQR), or frequency, n (%). Intraoperative complication denominators (hypoxia, bronchospasm) are 116, excluding one patient managed conservatively in the ICU without operation. ICU complications are expressed as a percentage of the 76 ICU-admitted patients; ARDS and HAP/VAP are expressed as a percentage of the full cohort (N = 117). ARDS: acute respiratory distress syndrome; HAP/VAP: hospital-acquired/ventilator-associated pneumonia; LOS: length of stay

Parameter	Variable	Value
Intraoperative complications	Hypoxia (SpO₂ <90%), n (%)	56 (48.3%)
	Bronchospasm, n (%)	22 (19.0%)
Immediate postoperative	Extubated on table, n (%)	43 (36.8%)
	Admitted to ICU, n (%)	76 (65.0%)
	Postoperative mechanical ventilation (days), median (IQR)	1 (0-4)
ICU complications	ICU hypoxia, n (%)	43 (56.6%)
	Therapeutic bronchoscopy required, n (%)	29 (38.2%)
Pulmonary complications	ARDS (Berlin definition), n (%)	28 (23.9%)
	HAP/VAP, n (%)	32 (27.4%)
Hospital course	ICU LOS (days), mean ± SD; median (IQR)	10.8 ± 13.4; 5 (2-15)
	Hospital LOS (days), mean ± SD; median (IQR)	20.0 ± 16.7; 13 (9-26)

Subgroup analysis: blunt vs penetrating injury

Patients with blunt injury were significantly older (median 40 vs 26 years; P < 0.001), had higher ISS (23 vs 16; P = 0.022) and TTSS (12 vs 10; P = 0.003), and underwent surgery later after injury (8 vs 5 days; P = 0.017). There were no significant between-group differences in OLV duration, peak lactate, or rates of intraoperative hypoxia, vasopressor use, or blood transfusion. The blunt injury group demonstrated a numerically higher ARDS rate (29.3% vs 11.4%; P = 0.057), significantly more frequent HAP/VAP (34.1% vs 11.4%; P = 0.013), and markedly longer ICU LOS (7 vs 3 days; P = 0.001) and hospital LOS (17 vs 10 days; P < 0.001) (Table 4).

**Table 4:** Subgroup analysis: blunt vs penetrating thoracic injury Data are presented as median (IQR) or frequency, n (%). * A p-value < 0.05 was considered statistically significant. Continuous variables were compared using the Mann-Whitney U test and categorical variables using the Pearson chi-square test, with Fisher’s exact test applied where any cell count was below five (no test statistic is defined for Fisher’s exact test). Intraoperative variables for the penetrating group are denominated on 34, excluding one nonsurgical patient. ARDS: acute respiratory distress syndrome; df: degrees of freedom; HAP/VAP: hospital-acquired/ventilator-associated pneumonia; ISS: Injury Severity Score; LOS: length of stay; OLV: one-lung ventilation; TTSS: Thoracic Trauma Severity Score

Parameter	Variable	Blunt (n = 82)	Penetrating (n = 35)	Test value (df)	p-value
Severity	Age (years), median (IQR)	40 (28-50)	26 (22-31)	U = 2200	<0.001*
	ISS, median (IQR)	23 (16-30)	16 (9-25)	U = 1814.5	0.022*
	TTSS, median (IQR)	12 (10-15)	10 (7-14)	U = 1932.5	0.003*
	Timing of surgery (days), median (IQR)	8 (5-13)	5 (1-10)	U = 1836	0.017*
Intraoperative	OLV duration (min), median (IQR)	41 (30-60)	48 (22-60)	U = 1476	0.809
	Peak lactate (mmol/L), median (IQR)	1.4 (1.0-2.0)	1.2 (0.8-2.2)	U = 1598.5	0.331
	Intraoperative hypoxia, n (%)	43 (52.4%)	13 (38.2%)	χ² = 1.94 (1)	0.163
	Vasopressor requirement, n (%)	26 (31.7%)	8 (22.9%)	χ² = 0.93 (1)	0.334
	Blood transfusion, n (%)	20 (24.4%)	6 (17.1%)	χ² = 0.75 (1)	0.388
Outcomes	ARDS, n (%)	24 (29.3%)	4 (11.4%)	Fisher’s exact	0.057
	HAP/VAP, n (%)	28 (34.1%)	4 (11.4%)	Fisher’s exact	0.013*
	ICU LOS (days), median (IQR)	7 (3-20)	3 (1-5)	U = 1985.5	0.001*
	Hospital LOS (days), median (IQR)	17 (9-30)	10 (6-14)	U = 2065.5	<0.001*

Subgroup analysis: OLV technique comparison

DLT had the longest median confirmation time (15 minutes, IQR 12-15) and EI the shortest (four minutes, IQR 3-5; Kruskal-Wallis H = 48.23, P < 0.001). OLV duration differed significantly across techniques (H = 19.58, P < 0.001), primarily reflecting case acuity and indication for surgery. First-technique failure was exclusive to the DLT group (8 of 59 DLT attempts failed and were rescued with a BB in seven cases and EI in one case). Once isolation was established, peak airway pressures were equivalent across techniques. Notably, intraoperative hypoxia rates differed significantly across techniques (DLT 37.3%, BB 57.8%, EI 66.7%; χ²(2) = 6.11, P = 0.047), a finding driven by case mix differences, as the BB and EI groups comprised more physiologically compromised patients undergoing damage control surgery. The same case mix confounding explains why DLT use was associated with the shortest hospital LOS (median 10 days) compared with BB (20 days) and EI (20 days; H = 12.63, P = 0.002) (Table 5).

**Table 5:** OLV technique comparison: DLT vs BB vs EI Data are presented as median (IQR) or frequency, n (%). * A p-value < 0.05 was considered statistically significant. Continuous variables were compared using the Kruskal-Wallis test; categorical variables using the Pearson chi-square test, with Fisher’s exact test applied where the expected cell count was below five (no test statistic is defined for Fisher’s exact test). Intraoperative hypoxia for the BB group is denominated at 45, excluding one nonsurgical patient. ARDS: acute respiratory distress syndrome; BB: bronchial blocker; df: degrees of freedom; DLT: double-lumen tube; EI: endobronchial intubation; LOS: length of stay; OLV: one-lung ventilation

Variable	DLT (n = 59)	BB (n = 46)	EI (n = 12)	Test value (df)	p-value
Confirmation time (min), median (IQR)	15 (12-15)	10 (7-14)	4 (3-5)	H = 48.23 (2)	<0.001*
OLV duration (min), median (IQR)	50 (35-65)	48 (30-68)	13 (12-18)	H = 19.58 (2)	<0.001*
Peak airway pressure (cmH₂O), median (IQR)	30 (26-35)	31 (27-36)	29 (27-36)	H = 1.59 (2)	0.451
Intraoperative hypoxia, n (%)	22 (37.3%)	26 (57.8%)	8 (66.7%)	χ² = 6.11 (2)	0.047*
ARDS, n (%)	9 (15.3%)	14 (30.4%)	5 (41.7%)	Fisher’s exact	0.061
Hospital LOS (days), median (IQR)	10 (7-18)	20 (10-35)	20 (8-33)	H = 12.63 (2)	0.002*

Subgroup analysis: VATS vs open thoracotomy

The groups were comparable in ISS, OLV duration, and intraoperative hypoxia rates. VATS patients had significantly lower peak lactate levels (1.1 vs 1.6 mmol/L; P = 0.037) and required intraoperative blood transfusion significantly less often (13.8% vs 31.0%; P = 0.026). The most clinically important finding was the substantially lower rate of ARDS in the VATS group (10.3% vs 36.2%; P = 0.001). There were additional nonsignificant trends favoring VATS: lower HAP/VAP (19.0% vs 34.5%; P = 0.059), shorter postoperative mechanical ventilation duration (one vs two days; P = 0.069), and shorter ICU LOS (four vs six days; P = 0.058). Hospital LOS did not differ significantly between groups (12 vs 18 days; P = 0.260) (Table 6).

**Table 6:** Subgroup analysis: VATS vs open thoracotomy Data are presented as median (IQR) or frequency, n (%). * A p-value < 0.05 was considered statistically significant. Continuous variables were compared using the Mann-Whitney U test and categorical variables using the Pearson chi-square test. One nonsurgical patient was excluded from this comparison. ARDS: acute respiratory distress syndrome; df: degrees of freedom; HAP/VAP: hospital-acquired/ventilator-associated pneumonia; ISS: Injury Severity Score; LOS: length of stay; MV: mechanical ventilation; OLV: one-lung ventilation; VATS: video-assisted thoracoscopic surgery

Parameter	Variable	VATS (n = 58)	Open (n = 58)	Test value (df)	p-value
Perioperative	ISS, median (IQR)	18 (16-25)	24 (16-28)	U = 1444	0.185
	Timing of surgery (days), median (IQR)	8 (5-11)	5 (0-15)	U = 2034.5	0.051
	OLV duration (min), median (IQR)	45 (34-60)	37 (18-60)	U = 1974	0.107
	Peak lactate (mmol/L), median (IQR)	1.1 (1.0-1.7)	1.6 (1.0-2.8)	U = 1303.5	0.037*
	Intraoperative hypoxia, n (%)	29 (50.0%)	27 (46.6%)	χ² = 0.14 (1)	0.710
	Vasopressor requirement, n (%)	12 (20.7%)	21 (36.2%)	χ² = 3.43 (1)	0.064
	Blood transfusion, n (%)	8 (13.8%)	18 (31.0%)	χ² = 4.96 (1)	0.026*
Outcomes	Extubated on table, n (%)	24 (41.4%)	19 (32.8%)	χ² = 0.92 (1)	0.336
	ICU admission, n (%)	34 (58.6%)	42 (72.4%)	χ² = 2.44 (1)	0.118
	ARDS, n (%)	6 (10.3%)	21 (36.2%)	χ² = 10.86 (1)	0.001*
	HAP/VAP, n (%)	11 (19.0%)	20 (34.5%)	χ² = 3.57 (1)	0.059
	Postoperative MV (days), median (IQR)	1 (0-3)	2 (0-5)	U = 1312.5	0.069
	ICU LOS (days), median (IQR)	4 (2-11)	6 (3-19)	U = 1339	0.058
	Hospital LOS (days), median (IQR)	12 (9-21)	18 (8-30)	U = 1477.5	0.260

Predictors of ARDS: logistic regression analysis

On univariate analysis, significant predictors of ARDS included ISS (OR 1.05; P = 0.014), TTSS (OR 1.15; P = 0.018), blunt injury pattern (OR 3.21; P = 0.046), preoperative mechanical ventilation (OR 5.72; P < 0.001), preoperative FiO₂ requirement (OR 1.03; P = 0.002), physiologically difficult airway (OR 3.36; P = 0.013), anatomically difficult airway (OR 2.76; P = 0.028), maximum intraoperative FiO₂ (OR 1.04; P = 0.002), vasopressor use (OR 2.81; P = 0.023), intraoperative blood transfusion (OR 3.19; P = 0.015), and intraoperative hypoxia (OR 2.68; P = 0.032). The VATS approach (OR 0.20; P = 0.002) and DLT vs EI (OR 0.25; P = 0.045) were protective. Age, tidal volume, PEEP, and peak airway pressure were not significant on univariate analysis.

Nine variables with univariate P < 0.20 or clinical relevance were entered into the multivariate model after excluding the one patient managed nonsurgically (final N = 116). The model demonstrated good discrimination (AUROC = 0.850) and calibration (Hosmer-Lemeshow P = 0.905), with a Nagelkerke R² of 0.415. After adjustment, the principal independent predictors of ARDS were preoperative mechanical ventilation (aOR 5.26; 95% CI 1.21-22.75; P = 0.026), maximum intraoperative FiO₂ (aOR 1.05 per percentage point; 95% CI 1.01-1.09; P = 0.021), and surgical approach, with VATS strongly protective relative to open thoracotomy (aOR 0.13; 95% CI 0.04-0.48; P = 0.002). Injury severity retained borderline significance (ISS aOR 1.05; 95% CI 1.00-1.11; P = 0.049). Peak lactate was inversely associated with ARDS in the adjusted model (aOR 0.56; 95% CI 0.35-0.90; P = 0.017), which may reflect healthy-survivor bias or preferential resuscitation of patients with higher lactate levels during surgery. The remaining variables, such as preoperative FiO₂, physiologically difficult airway, CPAP application, and intraoperative blood transfusion, did not retain significance after adjustment, suggesting that their univariate associations were mediated through shared covariates (Table 7).

**Table 7:** Univariate and multivariate logistic regression analysis for ARDS (N = 116) * A p-value < 0.05 was considered statistically significant. Associations were estimated by binary logistic regression; effect sizes are expressed as ORs with 95% CIs, and p-values were derived from the Wald test. Variables were entered into the multivariate model based on univariate P < 0.20 or strong clinical rationale; “Not entered” denotes variables excluded from the multivariate model. Model statistics: AUROC = 0.850; Nagelkerke R² = 0.415; Hosmer-Lemeshow goodness-of-fit P = 0.905. aOR: adjusted OR; AUROC: area under the receiver operating characteristic curve; CPAP: continuous positive airway pressure; DLT: double-lumen tube; EI: endobronchial intubation; ISS: Injury Severity Score; TTSS: Thoracic Trauma Severity Score; VATS: video-assisted thoracoscopic surgery

Variable	Crude OR (95% CI)	p-value	aOR (95% CI)	p-value
ISS (per unit)	1.05 (1.01-1.09)	0.014*	1.05 (1.00-1.11)	0.049*
TTSS (per unit)	1.15 (1.02-1.29)	0.018*	Not entered	-
Blunt (vs penetrating)	3.21 (1.02-10.08)	0.046*	Not entered	-
Preoperative mechanical ventilation	5.72 (2.28-14.36)	<0.001*	5.26 (1.21-22.75)	0.026*
Preoperative FiO₂ (per %)	1.03 (1.01-1.06)	0.002*	0.98 (0.93-1.02)	0.318
Physiologically difficult airway	3.36 (1.30-8.69)	0.013*	2.12 (0.58-7.71)	0.256
Anatomically difficult airway	2.76 (1.12-6.82)	0.028*	Not entered	-
Maximum intraoperative FiO₂ (per %)	1.04 (1.01-1.06)	0.002*	1.05 (1.01-1.09)	0.021*
CPAP to nondependent lung	2.76 (0.87-8.80)	0.086	1.39 (0.28-7.00)	0.690
Vasopressor requirement	2.81 (1.15-6.83)	0.023*	Not entered	-
Intraoperative blood transfusion	3.19 (1.25-8.17)	0.015*	2.70 (0.56-13.13)	0.218
Peak lactate (per mmol/L)	1.21 (0.98-1.50)	0.079	0.56 (0.35-0.90)	0.017*
Intraoperative hypoxia	2.68 (1.09-6.63)	0.032*	Not entered	-
VATS (vs open)	0.20 (0.07-0.55)	0.002*	0.13 (0.04-0.48)	0.002*
DLT (vs EI)	0.25 (0.07--0.97)	0.045*	Not entered	-

Correlation analyses

**Figure 2:**
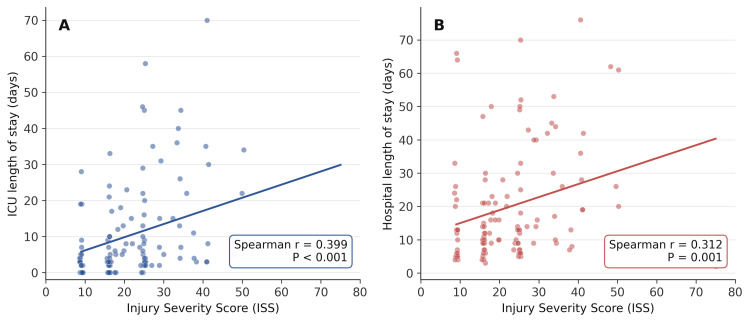
Spearman rank-order correlations between ISS and ICU and hospital lengths of stay ISS correlated positively with both ICU LOS (r = 0.399; P < 0.001) and hospital LOS (r = 0.312; P = 0.001). ISS: Injury Severity Score; LOS: length of stay

ISS demonstrated a statistically significant positive correlation with both ICU LOS (Spearman r = 0.399; P < 0.001) and hospital LOS (r = 0.312; P = 0.001), confirming the expected relationship between injury severity and healthcare resource utilization (Figure 2).

Peak lactate correlated positively with ICU LOS (r = 0.228; P = 0.013), supporting its utility as a marker of physiological derangement and resource intensity. OLV duration did not significantly correlate with intraoperative hypoxia (r = -0.018; P = 0.852), suggesting that hypoxia during OLV in this population is determined principally by patient physiological reserve and injury severity rather than the duration of lung isolation per se.

## Discussion

This retrospective observational cohort study describes the anesthetic management and outcomes of OLV in 117 consecutive thoracic trauma patients, representing one of the largest single-center series reported in this population. Several clinically important findings emerge. First, intraoperative hypoxia occurred in 48.3% of patients and varied significantly across lung isolation techniques (P = 0.047), with the lowest rate in the DLT group (37.3%) compared with the BB (57.8%) and EI (66.7%) groups. This difference is largely attributable to case mix confounding, as the latter techniques were preferentially employed in more physiologically compromised patients, indicating that the trauma milieu and patient selection, rather than the lung isolation device per se, drive the elevated hypoxia burden. Second, ARDS developed in nearly one-quarter of patients and was independently predicted by preoperative mechanical ventilation, maximum intraoperative FiO₂, and surgical approach, with VATS conferring striking protection relative to open thoracotomy. Third, blunt injury was associated with markedly worse ICU and hospital outcomes compared with penetrating trauma, reflecting higher injury burden rather than differences in anesthetic management. Taken together, these findings have direct implications for technique selection, ventilatory decision-making, and surgical planning in thoracic trauma.

The demographic profile of our cohort, predominantly young males sustaining blunt chest trauma from road traffic accidents, is consistent with published epidemiological data from comparable trauma registries. Large registry analyses confirm that blunt thoracic trauma constitutes the majority of thoracic injuries worldwide and is typically associated with higher ISSs and greater hemodynamic compromise than penetrating mechanisms [17,18]. The significant positive correlations we observed between ISS and both ICU LOS (Spearman r = 0.399; P < 0.001) and hospital LOS (r = 0.312; P = 0.001) align with established evidence that ISS correlates linearly with morbidity, hospital stay, and resource utilization [19].

The substantially higher ISS, TTSS, and longer hospital stay observed in blunt compared with penetrating injuries reflect the recognized biomechanical differences between these mechanisms. Blunt trauma, particularly from road traffic accidents, generates diffuse force vectors producing multisystem injury patterns, including rib fractures, pulmonary contusion, and hemopneumothorax, whereas penetrating injuries tend to be anatomically localized despite sometimes greater acute hemodynamic instability [18]. The TTSS correlated with injury severity in our series, supporting prior studies that proposed its utility for predicting outcomes in thoracic trauma [20]. The borderline difference in ARDS rates between blunt and penetrating injury (29.3% vs 11.4%; P = 0.057) most likely reflects the combined effects of pulmonary contusion, higher ISS, and greater preoperative ventilator dependence in blunt injury patients, all of which are established pathophysiological contributors to ARDS in the trauma setting.

DLTs were the most frequently used isolation technique in our series (50.4%), consistent with their recognized status as the gold standard for lung isolation in thoracic surgery [7-9]. A large systematic review of 39 randomized controlled trials reported a mean placement time advantage of 51 seconds for the DLT (95% CI 8-94 s; P = 0.02) and significantly lower malposition risk (OR 2.70; 95% CI 1.18-6.18) [9]. These findings are mirrored in our data, where eight of the 59 DLT attempts (13.6%) failed and required rescue (seven to a BB, one to EI); no failures occurred when a BB or EI was the first-chosen technique.

Despite these technical advantages, the appropriateness of DLT in acute trauma is often challenged by airway difficulty. Physiological difficulty was present in 53.8% and anatomical difficulty in 26.5% of our patients, rates far exceeding those in elective thoracic surgery series. In these circumstances, BBs offer the practical advantage of placement through an existing single-lumen tube, avoiding the cardiovascular perturbation of an additional laryngoscopy [7]. A propensity-matched analysis of 4,636 elective thoracic resection patients found that BB use was associated with greater postoperative pulmonary infiltrates and longer ICU stay than DLT, though this likely reflects case mix confounding rather than a causative device effect [21]. In our series, the longer hospital LOS in BB patients vs DLT (20 vs 10 days; P = 0.002) most plausibly reflects the preferential use of BBs in hemodynamically compromised damage control surgery cases.

EI accounted for 10.3% of cases and was confirmed in a median of four minutes (IQR 3-5), significantly faster than either DLT or BB, making it the technique of choice for emergent damage control procedures in our center. Although intraoperative hypoxia rates differed significantly across techniques (DLT 37.3%, BB 57.8%, and EI 66.7%; χ²(2) = 6.11, P = 0.047), this gradient closely tracked case mix rather than device performance, as BBs and EIs were preferentially deployed in more physiologically compromised, damage control patients. Peak airway pressures did not differ across techniques, supporting the principle that all three techniques can achieve safe lung isolation when selected appropriately to the clinical context.

Intraoperative hypoxia (SpO₂ <90%) occurred in 48.3% of our patients. This elevated rate is physiologically expected in the trauma context. Preoperative models incorporating physiological and procedural variables have been proposed to risk-stratify hypoxemia during OLV and may help anticipate intraoperative desaturation in high-risk patients [22]. Preexisting hemodynamic instability, pulmonary contusion, hemopneumothorax, and ongoing hemorrhage all impair hypoxic pulmonary vasoconstriction, the principal physiological mechanism that redirects blood flow from the nonventilated lung and limits the obligate shunt during OLV [23]. The absence of a significant correlation between OLV duration and intraoperative hypoxia (r = -0.018; P = 0.852) suggests that hypoxia was driven by an underlying physiological state rather than the duration of lung isolation.

ARDS developed in 23.9% of our patients, substantially higher than the 1.2-4.2% reported in elective pulmonary resection studies but consistent with the known pathophysiology of post-traumatic ARDS, in which lung contusion, systemic inflammation, transfusion-associated lung injury, and VILI act synergistically [24]. Our multivariate model identified three independent predictors of ARDS: preoperative mechanical ventilation, maximum intraoperative FiO₂, and open thoracotomy. Preoperative mechanical ventilation was the strongest independent predictor of ARDS (aOR 5.26; 95% CI 1.21-22.75; P = 0.026). This is consistent with prior evidence that patients already receiving positive-pressure ventilation at the time of surgery carry both greater baseline lung injury and cumulative iatrogenic VILI exposure [24]. These patients are physiologically primed for further lung injury by the combined insults of preexisting pulmonary inflammation, surgical manipulation, the obligate shunt of OLV, and reperfusion injury at lung re-expansion. Targeting preoperative ventilatory parameters, particularly tidal volume, PEEP, and driving pressure, in this subgroup represents a modifiable target for ARDS prevention even before induction of anesthesia.

Maximum intraoperative FiO₂ was another independent ARDS predictor (aOR 1.05 per percentage point; P = 0.021). Although this may partly reflect reverse causality, with higher FiO₂ administered in response to worsening oxygenation, there is a strong biological rationale for FiO₂-mediated lung injury. Hyperoxia promotes oxidative stress, absorption atelectasis, and pro-inflammatory cascades that potentiate ARDS pathogenesis [1]. Current consensus guidelines recommend minimizing FiO₂ to maintain SpO₂ ≥90%, rather than targeting normoxia or hyperoxia [25]. In the trauma OLV setting, where the inclination to administer high FiO₂ is understandable, measured restraint in FiO₂ escalation, with preferential use of CPAP, PEEP optimization, and temporary restoration of two-lung ventilation, may confer meaningful lung-protective benefit.

The most striking finding of this study was the independent protective effect of VATS against ARDS. After adjustment for injury severity, preoperative ventilation, and other confounders, VATS was associated with an aOR of 0.13 (95% CI 0.04-0.48; P = 0.002) relative to open thoracotomy, corresponding to an 87% relative reduction in ARDS odds. The unadjusted ARDS rates (10.3% vs 36.2%; P = 0.001) reinforce this finding. VATS patients also had significantly lower intraoperative blood transfusion requirements (13.8% vs 31.0%; P = 0.026) and lower peak lactate levels (1.1 vs 1.6 mmol/L; P = 0.037), consistent with less hemodynamic derangement and surgical trauma. Open thoracotomy disrupts chest wall mechanics through rib spreading, intercostal nerve injury, and diaphragmatic retraction, all of which impair postoperative respiratory mechanics and predispose to atelectasis, secretion retention, and ventilator dependence. VATS, by contrast, preserves chest wall integrity, reduces the inflammatory response to surgical trauma, and facilitates earlier extubation [4,5]. However, given the observational design and the possibility of residual confounding, this association should be interpreted cautiously and confirmed in prospective studies before definitive recommendations regarding surgical approach can be made.

A uniform lung-protective strategy was applied throughout our cohort, with a median tidal volume of 5 mL/kg predicted body weight, PEEP of 5 cmH₂O, and peak airway pressures of 30 cmH₂O. This is consistent with international consensus recommendations for OLV, which advise tidal volumes of 4-6 mL/kg predicted body weight, individualized PEEP to minimize driving pressure, and avoidance of high FiO₂ as a routine rescue maneuver [25]. A 2026 multicenter registry-based analysis confirmed that adherence to protective ventilation parameters during thoracic surgery was associated with reduced PPCs [26]. In trauma patients, where the physiological margin for error is narrowed by hemodynamic instability, coagulopathy, and concurrent lung injury, adherence to low-tidal-volume, driving-pressure-limited ventilation is particularly critical. Driving-pressure-oriented ventilation, in which PEEP is individually titrated to minimize the pressure difference between plateau and PEEP, has demonstrated improved PaO2/FiO₂ ratios and reduced PPCs vs fixed-parameter strategies in a systematic review and meta-analysis of seven studies totaling 640 patients [27].

HAP/VAP developed in 27.4% of our patients and was significantly more common in blunt injury patients, consistent with the longer mechanical ventilation duration and ICU stay associated with blunt thoracic trauma. Rates were numerically higher in the open thoracotomy group (34.5% vs 19.0%; P = 0.059), likely reflecting the impaired respiratory mechanics, greater chest wall pain, and longer ventilator dependence following thoracotomy. Epidural analgesia was employed in 39.3% of our patients and ESPB or SAPB in 15.4%, reflecting an increasing institutional trend toward opioid-sparing regional anesthesia. These approaches facilitate deep breathing, effective coughing, and early mobilization and are central to enhanced recovery pathways in thoracic surgery.

Strengths and limitations

This study has several strengths. It includes all consecutive trauma patients requiring OLV identified from a high-volume Level I trauma center registry over six years, without selection or exclusion bias, and encompasses a broad range of injury patterns, mechanisms, and OLV techniques. The sample size of 117 is among the largest reported specifically for OLV in the trauma context. The logistic regression model was rigorously constructed with formal calibration testing, yielding strong discrimination (AUROC = 0.850) and good calibration (Hosmer-Lemeshow P = 0.905).

Several limitations must be acknowledged. First, the retrospective single-center design is inherently susceptible to information bias, residual confounding, and limited generalizability to centers with different trauma case mixes, anesthetic expertise, or surgical resources. Second, the selection of lung isolation devices, ventilatory strategies, rescue oxygenation measures, and surgical approaches was determined by treating clinicians rather than standardized institutional protocols, introducing potential treatment-selection bias and practice variability. Third, although only 15 of 132 screened patients were excluded, the use of complete-case analysis without imputation may have introduced selection bias because patients with missing key ventilation or intraoperative data were excluded from analysis. Fourth, only 28 patients developed ARDS, which may limit the stability of multivariable regression estimates and raises the possibility of model overfitting despite careful variable selection and acceptable model performance metrics. Fifth, unmeasured confounders, including surgeon preference, evolving institutional practice patterns over the six-year study period, and factors influencing surgical decision-making, could not be fully accounted for. Finally, although VATS remained independently associated with a lower incidence of ARDS after adjustment, residual confounding by indication cannot be excluded because patients selected for minimally invasive surgery may have differed in important clinical characteristics not fully captured by measured covariates; therefore, the observed protective association of VATS should be interpreted as hypothesis-generating rather than evidence of causality.

## Conclusions

OLV in thoracic trauma is associated with a substantial burden of intraoperative hypoxia and PPCs, including ARDS. The choice of lung isolation device was not independently associated with ARDS, supporting individualized device selection based on patient characteristics, airway considerations, and clinical context. Preoperative mechanical ventilation, higher intraoperative FiO₂ requirements, and surgical approach were independently associated with ARDS, whereas VATS was associated with a lower incidence of ARDS than open thoracotomy. These findings identify potentially modifiable perioperative factors and provide a foundation for future prospective studies aimed at optimizing outcomes in thoracic trauma patients requiring OLV.
